# Aggravation of Obsessive-Compulsive Disorder Due to Excessive Porn Consumption: A Case Report

**DOI:** 10.7759/cureus.33018

**Published:** 2022-12-27

**Authors:** Tejas Shrivastava, Pratik Agarwal, Vidhi Vora, Yashendra Sethi

**Affiliations:** 1 Medical Officer, Community Health Center (CHC) Nikum, Durg, IND; 2 Medicine, Lokmanya Tilak Municipal Medical College and General Hospital, Mumbai, IND; 3 Internal Medicine, Government Doon Medical College, Dehradun, IND

**Keywords:** behavioral disorder, porn addiction, obsessive compulsive disorders, ocd, harmful effects of porn, excessive porn consumption, behavioral addiction, pornography 2022

## Abstract

The past few decades have seen a significant rise in pornography consumption. This has brought into existence a new behavioral addiction, addiction to internet pornography, which impacts the psycho-somatic health of the individuals and people around them. The accessibility, affordability, and anonymity of online pornography have fed the growing popularity of online pornography. The International Classification of Diseases (ICD-11) has included pornography in Compulsive Sexual Behavior Disorder with it being categorized as an impulse control disorder and not necessarily an addictive disorder. However, the recently published Diagnostic and Statistical Manual of Mental Disorders, Fifth Edition, Text Revision (DSM-5-TR) does not recognize a diagnosis of sexual addiction/compulsion (including internet pornography). Psychiatry remains an ever-evolving branch, and as the understanding evolves, the schools of thought change as well. The constantly evolving literature on behavioral addictions has helped the understanding that any source capable of stimulating the reward circuitry in an individual can become addictive. The stigma associated with behavioral addictions, particularly pornographic addiction, as well as a lack of awareness, contribute to under-reporting, making the reported cases just the tip of the iceberg. Addictions have been associated with various complications and other psychiatric phenomena. We describe a case of a 28-year-old male with mild features of obsessive-compulsive disorder (OCD) that took major shape with the advent of pornographic addiction. The patient was a known case of exam anxiety, panic attacks, auditory hallucinations, and mild obsessive and compulsive symptoms. However, with the death of his girlfriend’s father, a breakup with his girlfriend, and coronavirus disease 2019 (COVID-19), he indulged in porn consumption which soon shifted to religious pornographic content leading to guilt with a fair insight. This later culminated in the aggravation of his OCD with repetitive cleaning activities. The patient was started on pharmacological and behavioral therapy and has responded well in follow-ups. In light of the special presentation of this case, we strongly recommend better reporting of such complications aiding in broadening the understanding of the spectrum of possible psychiatric impacts of pornographic consumption.

## Introduction

The use of pornography has significantly increased during the last few decades. Addiction to internet pornography has emerged as a new type of behavioral addiction. The rising popularity of internet pornography has been fueled by its accessibility, affordability, and anonymity [1]. The Compulsive Sexual Behavior Disorder (CSBD) in the International Classification of Diseases (ICD-11) now includes pornography, which is classified as an impulse control disease rather than necessarily an addiction condition [2]. The Diagnostic and Statistical Manual of Mental Disorders, Fifth Edition, Text Revision (DSM-5-TR), released in March 2022, however, does not recognize the diagnosis of sexual addiction or compulsion (including internet pornography). Psychiatry, being an ever-evolving discipline, sees a regular growth in understanding and the accepted school of thought. Continuous development has made it easier to recognize that any source capable of stimulating an individual can contribute to behavioral addictions. Addiction is defined by DSM-5 as a chronic, relapsing disorder characterized by compulsive drug seeking and use despite adverse consequences. The expanding neuro-scientific research in this area has helped us understand its mechanisms: Regardless of the diversity of chemicals, all the drugs of abuse usually affect a similar circuitry in the limbic area and relay their rewarding effects [3]. Repeated exposure to the stimuli is also said to cause a permanent change in the neural circuitry, thus explaining the chronic course of addiction [4]. Behavioral addiction, in a similar way, operates through the reward circuitry [3-6]. With the phenomenal rise in internet use in the past few decades, the use of pornography has risen manifolds, thus bringing into existence a new behavioral addiction, addiction to internet pornography. Porn addiction or "problematic pornographic use" is said to affect around 3-6% of the entire population [6]. Pornographic consumption causes a release of dopamine within the limbic reward system, causing neuroplastic changes in the pathway and thus perpetuating the experience. These changes gradually get integrated as "brain maps" for sexual excitement. Researchers have also alluded to the concept of tolerance as a result of repeated internet pornography consumption, which leads to the individual consuming more graphic content [7].

The progressive addiction to internet pornography has an impact on the psycho-somatic health of the individuals and people around them [6]. Compared to healthy controls or moderate porn users, frequent internet pornography viewers need more visual stimulation to trigger brain responses [8]. When making decisions, sexual desire might obstruct the ability to receive feedback and make wise choices [9]. Similar to this, sexual arousal brought on by sexual pictures was found to temporarily reduce working memory performance [10]. Studies have reported a decrease in executive performance in hypersexual individuals [11]. A significant impact was also reported on the partners in terms of a sense of betrayal, disconnection, and trauma-like experience [12]. The constantly evolving literature on behavioral addictions has aided in the development of the understanding that any source capable of stimulating an individual can become addictive. An increased level of internet addiction has been associated with significant functional impairment in terms of loneliness, depression, and compulsion [13]. However, the stigma associated with behavioral addictions, particularly pornographic addiction, as well as a lack of awareness and reporting, contribute to under-reporting, hence leading to the hidden burden of this addiction. 

Behavioral addictions differ in being typically ego-syntonic, in contrast to ego-dystonic obsessive-compulsive disorder (OCD). However, both OCD and internet addiction score highly on harm avoidance measures. When compared to other behavioral addictions, compulsivity is more prominent in OCD. Psychological interventions for behavioral addictions focus on avoidance and relapse prevention, whereas those for OCD focus on exposure and response prevention. The current literature has scanty data on the association between the two or the co-existence of both these disorders. We present an interesting case report of a patient with co-existence of excessive pornographic consumption and OCD.

## Case presentation

Clinical course

A 28-year-old male of middle socioeconomic status from an urban background presented with the complaint of inability to forget certain dates and times for the last three years, an association of negative thoughts to unrelated objects for the last 1.5 years, a sense of disgust from self during certain activities since the last 1.5 years, a disturbing association of days and dates with memories and avoiding them since the last 1.5 years and repetitive cleaning and hand-washing behavior associated with disgust since one year.

The patient was apparently alright around four years back when one of the usual exam panics turned more intense. He used to have exam-related anxiety for the past few years due to the professional nature of his course. However, four years back, in a solitary incident of exam anxiety, he felt he heard his voice in his internal space laughing at him and saying that he cannot do it. He also had a single isolated incident of what he described as a panic attack. He noticed his breath become fast and shallow and his heart pounding. For one week after this episode, he expressed fear of forgetting basic skills such as language, drinking water, and walking. Despite the wish and effort to forget certain things, he could not get over them: he could not forget the specifics of the death of his girlfriend's father, three years back. The same year he had a fight with his friend and couldn't forget the date and time of the same. Later that year, his girlfriend broke up with him. He developed a habit of remembering dates of such bad incidents including the death of two other neighbors at the end of that year who he was merely acquainted with. Two years back, he learned about the death of his best friend’s grandfather who he had been closely caring for. The patient could not forget that date and time. The same afternoon he had bought a few notebooks that he never used after that, with a thought that something bad would happen if he used them. He could not forget the dates of death of other family members too which he gradually started to avoid. 

Last year, during the coronavirus disease 2019 (COVID-19) lockdowns, he also started using unconventional genera of pornography (involving religious figures) which began as a curiosity, with one to two videos once a week but gradually turned into a habit. Within three months, he started using one to two videos daily and reached a phase where conventional porn did not have the same effect. He gradually developed a sense of guilt and the notion that his mind was contaminated. He tried to cut off his internet usage but could not forget the images and would often visit back to the websites, unable to limit the use. He developed an irresistible urge to visit back to consume porn which was stimulated with each attempt to abstain from it. He reported that the withdrawal was associated with irritation, anxiety, and inability to sleep. Consumption of porn had become his escape from troubles and stress in life. During the same time, he also started avoiding any activity of importance on the dates and times of negative events that he had in his memory. He developed a sense of disgust that his mind was "polluted". He started extensive repetitive and insatiable cleaning, which probably developed to counter the guilt.

**Figure 1:**
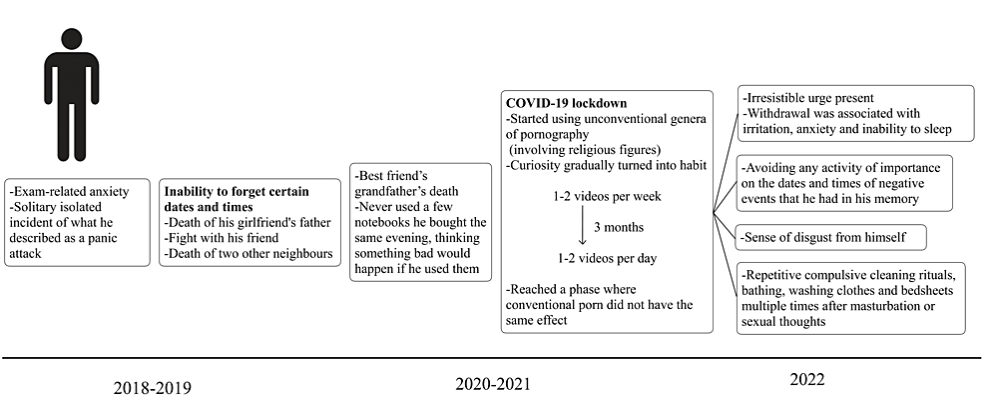
Timeline of events

He made attempts to neutralize his sexual thoughts through repetitive compulsive cleaning rituals, bathing, and washing clothes and bedsheets multiple times after masturbation or sexual thoughts. The mental status examination (Table 1) reported an apprehensive mood and congruent anxious affect. His thought content consisted of obsessive rumination, the compulsion of washing, and feelings of guilt with fair insight. The thoughts were severe and intrusive, ego-dystonic, causing significant distress, day-to-day dysfunction, and socio-occupational impairment. The family understood the disorder and encouraged the patient to seek professional help. The timeline of events is summarized in Figure 1.

**Table 1:** Mental Status Examination (MSE)

MSE Component	Reported findings
General appearance and Behavior	The patient was well-groomed and dressed appropriately. Psychomotor activity was normal. Eye contact was established and maintained, and rapport was easily established. He was cooperative and had no tics or mannerisms.
Speech	The patient's speech was spontaneous, and audible, with normal reaction time and pitch. Appropriate tonal variation was present. The speech was relevant and coherent, the answers were appropriate and understandable.
Mood	The patient expressed anxiety and appeared anxious. Reactivity was present with a restricted range. His mood was appropriate for the situation and congruent with thought. There was no liability.
Thought	Stream: Normal, goal-directed thought with no thought block, circumstantiality, tangentiality, perseveration, flight of ideas or stereotypy.
	Form: Well-structured, understandable form with no loosening of association, poverty of production or content of speech.
	Possession: Recognized his thoughts as his own. Obsessions about porn consumption and compulsions about cleaning rituals were present.
	Content: Preoccupation with catastrophizing, guilt and shame.
Perception	The patient had no perception anomalies such as hallucination, illusion, derealization or depersonalization.
Attention	The patient could perform the digit forward, and digit backward tests well. His attention was appropriate and concentration adequate.
Orientation	Time: The patient could tell the time of the day, date, month of the year.
	Place: He could tell where he was and the name of the town.
	Person: He gave his name, age, occupation and other details correctly; he correctly identified the doctor, nurse, and his attendant
	The patient was well-oriented to time, place and person
Memory	Immediate: The patient had no difficulty in doing the digit forward and digit backward tests
	Recent: He remembered correctly what he had for breakfast and how he arrived at the hospital.
	Remote: He remembered the timeline of his education correctly.
	His memory was intact.
Intelligence	General fund of knowledge: The patient was aware and informed about the current who's-who and major events
	Mathematical ability: Simple and carryover calculations were easily performed. Abstraction: He knew the similarities and differences between a chair and a table, a fan and a lightbulb. Abstract meanings of proverbs were correctly explained.
	His intelligence was appropriate.
Judgement	Test: On being asked a question as to what he would do if his house caught fire, he said that he would call the fire department (fire brigade) for help and try putting it out
	Social: He was well-behaved in the department.
	Personal: On being asked about his future plans, he mentioned his plans for completing his education and getting a job.
	His judgement was intact.
Insight	The patient recognized that he had an illness at an intellectual level, the knowledge of which wasn't applied. His level of insight was 4.

Diagnostic assessment

To diagnose the case, DSM-5 was used as the basis. The presence of obsession over religious pornographic content causing evident distress along with attempts to ignore and suppress these thoughts was seen. As the current version of DSM-5 doesn't have a specific categorization for internet use disorder and pornography addiction, the criteria for Internet Gaming Disorder (IGD) was modified following other substance use disorders. Analyzing the case through this, made it clear that the patient was preoccupied with porn consumption, spending a significant amount of time either consuming porn or thinking about the same, with an increased requirement for content gradually. With every attempt to abstain from it, he became anxious and irritable and experienced disturbed sleep. While this use was causing him significant distress and dysfunction, all attempts to cut down on it were unsuccessful. He had intense cravings to consume porn which caused recurrent relapses. A provisional diagnosis of OCD with fair insight and internet use disorder was made based on this.

Treatment course

Pharmacological Management

We started the patient with a selective serotonin reuptake inhibitor (SSRI) as the first-line treatment, in order to deal with the serotonergic stimulatory and reinforcement behaviors. Capsule fluoxetine 20 mg once daily was started in the morning and gradually increased by 20 mg each week to a dose of 60 mg over a period of three weeks. This was continued for 10 months and no augmentation was required. Tab clonazepam 0.5mg one tablet at night was prescribed for 10 days then the patient was allowed to use it only on days he had excess nervousness.

Psychological Intervention

**Figure 2:**
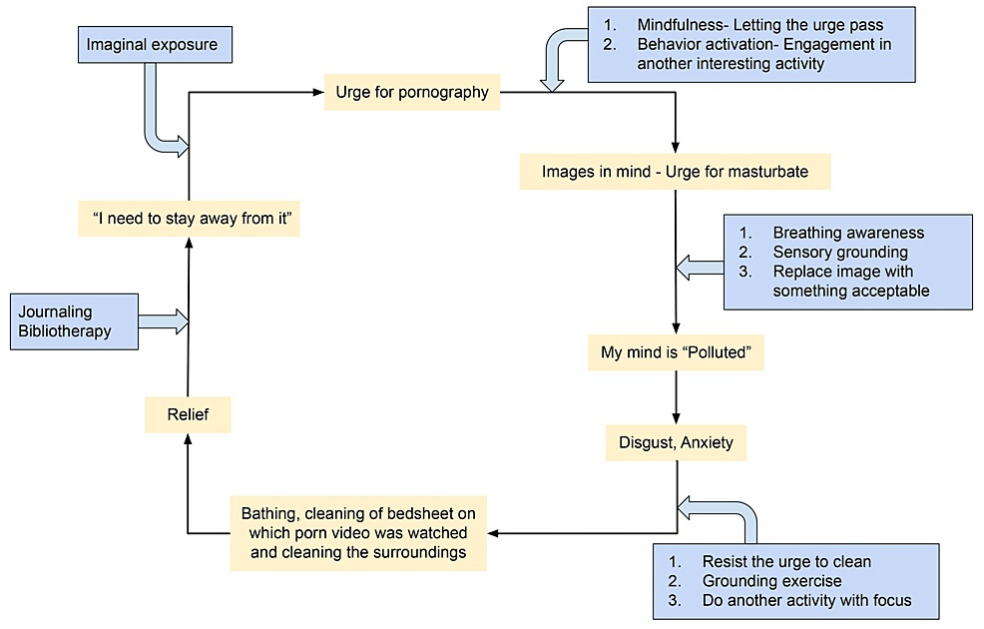
Multi-dimensional de-addiction therapy targeting various components of the patient’s disease.

Psychoeducation regarding the disorder, medicines, and expected course of treatment was also provided to the patient. A blend of Rational Emotive Behavior Therapy (REBT) and Cognitive Behavioral Therapy (CBT) was applied using Socratic questioning and an outline map of behavior sequence and patterns was made, triggers identified and appropriate alternatives were explored. A plan of action was formulated to incorporate healthy activities, socialization, and hobbies as an alternative to internet use. Information regarding Exposure and Response Prevention was provided, and sessions were conducted with imaginal exposure to remove associated compulsive behaviors. The steps of Motivational interviewing were applied in each session along with introducing and following up steps for relapse prevention. As a part of psychotherapeutic management, we first addressed the "urge to watch pornography" by employing mindfulness-based practices and instructing the patient to focus on the small specifics of his daily activities while engaging all of his senses, and "being in the present". Similarly, an urge to masturbate was diverted by teaching him various sensory grounding techniques that he could use whenever an unpleasant urge arose. The primary focus was on replacing the recurring urge with other acceptable activities as listed by the patient. Since the blasphemous content of the particular pornography triggered his compulsive patterns, we concentrated on redirecting his attention to other acceptable forms of arousal. The thought "My mind is polluted," followed by an incessant urge to clean, was then focused on in therapy. When such thoughts arose, he was guided to observe each thought and sensation that arose before acting on it. A metaphorical explanation of treating thoughts as "waves" that come and go was given, which aided him in the process. Simultaneously, he was journaling any insights and breakthroughs for later reference to prevent relapse. He was given information in the form of psychoeducation and information booklets with an emphasis on him not being alone. The patient was thus treated in a well-calculated way targeting various components of his disease (Figure 2).

Outcome

The patient showed significant improvement in behavior patterns related to internet use disorder. The need for frequent follow-ups has been reduced to once a month for relapse prevention. The severity of daily distress and socio-occupational dysfunction have also improved significantly with the patient now able to continue his education and attend work.

## Discussion

Addictions are associated with neurochemical changes in the mesolimbic dopaminergic pathway including the ventral tegmental area and the nucleus accumbens [3]. This is often associated with hypofrontal states in the orbitofrontal cortex, which may contribute to impulsivity, emotional lability, compulsion, and impaired judgment [14]. A similar effect of change in the cellular architecture of the nucleus accumbens has been shown to occur with sexual behavior as pointed out by Pitchers et al. [15]. A study on compulsive sexual behaviors (CSB) found that CSB persons had a larger urge to watch sexually explicit movies than controls, but a lesser liking to those same videos. Similar studies examining the activity of common brain areas during drug-craving states revealed that CSB participants had higher levels of the regions' activity when exposed to sexually explicit content [8]. Brown et al. laid down the criteria to consider an activity addictive which includes salience, euphoria, tolerance, withdrawal symptoms, conflict, and relapse. The DSM-5-TR, released in March 2022, does not recognize a diagnosis of sexual addiction/compulsion that includes internet pornography. This, however, is classified as an impulse control disorder rather than an addictive disorder. Hypersexual disorder, which includes excessive pornographic consumption has however been included in DSM-5 [16]. Pornography use has been added to CSB disorders in ICD-11. Regardless, several studies have linked the effects of compulsive pornography viewing and other behavioral addictions to substance addiction, paving the way for them to be possibly included in DSM-5 in the future [3,6,7,16].

Behavioral addictions, despite being a separate entity, have some overlaps with OCD. This was seen in this reported case where the patient’s OCD either came to light due to his pornographic addiction or was perpetuated as a result of the same. This scenario supports the behavioral addiction model of obsessive-compulsive disorder as pointed out by Grassi et al. [17]. They assessed three dimensions of OCD and normal subjects using the Barratt Impulsiveness Scale, Iowa Gambling Task, and Beads Task. Patients with OCD were concluded to be more impulsive than controls, exhibiting risky decision-making and biased probabilistic reasoning. Similarly, Rai et al. compared behavioral addictions and OCD in their article and showed a significant prevalence of internet and pornographic addiction (p<0.001) in subjects with OCD as compared to controls. Subjects with coexisting behavioral addictions and OCD were also found to rate higher on impulsivity, motor, and attention sub-scales [18]. Pornography sites are specifically designed to perpetuate the self-reinforcing chemical-induced cycle thereby proceeding toward addiction. This, combined with the taboo surrounding its discussion, results in a significant "hidden burden" of this problem, which has an impact on personal, relationship, professional, and organizational levels [12].

Pornographic consumption is difficult to uncover due to the taboo associated with it. However, the fact that this patient sought therapy for an addiction to the same indicates the likely distress it caused him. SSRIs were among the first drugs used to treat behavioral addictions. CBT is utilized for the control of OCD, either alone or in combination with SSRIs as the first line of management. In this case, SSRIs were used in conjunction with CBT and REBT as the modality of management, and considerable improvement was seen. This could allude to the use of this modality in such comorbid conditions.

Many psychiatrists in their work have highlighted the need for public health intervention and legislation to serve the cause. To cite an example, Sharpe et al. listed down a few important health policy considerations including public health campaigns, school education, age verification, and precautionary principles to help curb the problem [19].

## Conclusions

The discussed case is a prime example that psychiatric manifestations usually co-exist and often are associated. The case had an interesting presentation showing aggravation of OCD symptoms after excessive porn consumption, underlining the need for detailed history and examination in such patients and having a degree of suspicion. The association between behavioral addictions, especially pornography use, and OCD points to an intriguing model of the development of OCD that merits additional investigation and can help define the disease's future path of treatment. It is also fairly evident from this case that porn addiction can have long-lasting effects on mental health which cannot only create but also aggravate psychiatric illnesses. We also feel that there is a need for public health intervention for combating the same in terms of increased awareness, which will facilitate increased reporting and recognition of the problem by the relevant authorities. The only way to promote early diagnosis and intervention is to work against the taboo. We advocate for improved telemedicine and e-psychiatry resources to assist people in seeking psychiatric help. A national mental health helpline can also help better accessibility. There is a pressing need for more research in this area to help structure the possible classification of behavioral addictions, particularly pornographic addictions, thus guiding the policy and treatment in the future.
